# Vaccination for SARS-CoV-2 of migrants and refugees, Jordan

**DOI:** 10.2471/BLT.21.285591

**Published:** 2021-09-01

**Authors:** Saverio Bellizzi, Chinara Aidyralieva, Lora Alsawhala, Ala’a Al-Shaikh, Alessio Santoro, Maria Cristina Profili

**Affiliations:** a World Health Organization Country Office, Mohammad Jamjoum Street Ministry of Interior Circle, Building 5, 11181 Amman, Jordan.

By 14 January 2021, Jordanian authorities had reported almost 320 000 cases of coronavirus disease 2019 (COVID-19) and more than 4000 deaths due to the disease. These figures correspond to about 6.0% and 3.0%, respectively, of the morbidity and mortality in the World Health Organization’s (WHO) Eastern Mediterranean Region.^1^ On the same date, Iraqi and Syrian refugees living in camps received their first dose of COVID-19 vaccine in a public health clinic, making Jordan an example of equitable access to health life-saving care.^2^

The Jordanian government faces several challenges in responding to the COVID-19 pandemic due to the country’s demographics. The International Organization for Migration reports that four in 10 people living in Jordan are immigrants.^3^ According to the United Nations High Commissioner for Refugees (UNHCR), 658 000 Syrian refugees are registered in the country;^4^ however, the United Nations Children’s Fund estimates that the total number is rather around 1.3 million, with most living out of camps.^5^ On the other hand, more than 2 million registered Palestine refugees live in Jordan, with about 18.0% (370 000) of these hosted in 10 recognized camps throughout the country.^6^ Jordan also hosts refugees from other countries of origin (67 000 Iraqis, 15 000 Yemenis and 6000 Sudanese, among others).^7^

Since the first case of COVID-19 among refugees was confirmed in the country in September 2020, 1928 refugees living in refugee camps serviced by UNHCR have tested positive for the disease, with the proportion of positive cases remaining low, at 1.6% (1928/120 000) in UNHCR camps, compared to 3.0% (320 000/10 300 000) among the general Jordanian population in January 2021.^2^

At the onset of the outbreak, the health ministry supported by WHO rapidly produced the National Preparedness and Response COVID-19 Plan. The plan emphasized a whole-of-society approach, with beneficiaries including Jordanians and non-Jordanians residing in both host communities and refugee camps. The national plan was also the overarching framework for the following plans targeting specific populations: UNHCR’s Jordan Refugee Response Coronavirus Contingency and Response Plan and the United Nations Relief and Works Agency for Palestine Refugees in the Near East’s COVID-19 Strategic Preparedness and Response Plan. The Jordan COVID-19 National Deployment and Vaccination Plan followed in December 2020 and was conceived to further extend free-of-charge equitable access of all individuals in Jordan.

About 85.0% of the Syrian refugee population in Jordan (571 572/672 438) was highly or severely vulnerable in 2018, living below the poverty line.^4^ To give vulnerable populations, such as migrants and refugees,^8^ priority during the planning of vaccination campaigns is often politically untenable. However, by applying the concept of health systems inclusiveness and equity with the rest of the population, the Jordanian government has made a critical step forward towards universal health coverage and rights to health in general. Since the beginning of the pandemic, the government had indicated its intention to use the same prioritization criteria (health-care workers, older people and people with underlying health conditions) for all, irrespective of nationality and/or residency status.

However, previously published reports^9^ point out that implementation of such planning is hard because of challenges such as providing vaccination for hard-to-reach refugees and migrants – including irregular migrants who would normally be reluctant to access services. In this regard, the Jordanian health ministry is actively involved in tailoring public communication and awareness campaigns.

While the efforts in Jordan to provide COVID-19 vaccination among refugees is commendable, the pandemic will only end if diagnostic and treatment tools as well as vaccines are shared equitably.

The world’s population has so far had inequitable access to vaccines; Jordan can set an example for other countries to follow. 
